# Environmental Circulation of *Aspergillus fumigatus* With Reduced Susceptibility to Agricultural Triazole in Brazil: Clonal Dissemination of Potentially Resistant Genotypes

**DOI:** 10.1111/myc.70179

**Published:** 2026-04-27

**Authors:** Dality Keffelen de Barros Rodrigues, Manuel Leeuwerik, Balázs Brankovics, Wellington Santos Fava, James Venturini, Wieland Meyer, Teppei Arai, Hidetaka Majima, Akira Watanabe, Marcia de Souza Carvalho Melhem

**Affiliations:** ^1^ Federal University of Mato Grosso do Sul Campo Grande Brazil; ^2^ Westerdijk Fungal Biodiversity Institute Utrecht the Netherlands; ^3^ Hogeschool Utrecht Utrecht the Netherlands; ^4^ Sydney School of Veterinary Science The University of Sydney Sydney New South Wales Australia; ^5^ Curtin Medical School Curtin University Perth Western Australia Australia; ^6^ Fundação Oswaldo Cruz Rio de Janeiro Brazil; ^7^ Medical Mycology Research Center Chiba University Chiba Japan; ^8^ São Paulo State University Botucatu Brazil; ^9^ Lim 53 Laboratory of Medical Investigation, School of Medicine University of São Paulo São Paulo Brazil

**Keywords:** antifungal drug resistance, CRISPR‐Cas systems, CYP51 enzymes, microsatellites, tebuconazole, whole genome sequencing

## Abstract

**Background:**

*Aspergillus fumigatus* resistance to triazole antifungals poses an increasing global health concern. Moreover, the cross‐resistance between azole antifungal agents used in clinical settings and those applied in agriculture has become an important emerging issue.

**Objectives:**

In this study, we investigated the five environmental *A. fumigatus* strains showing reduced susceptibility to tebuconazole.

**Methods:**

Fungal strains were recovered from air samples collected around the homes of two patients with suspected aspergillosis caused by resistant isolates. Species identification was performed by sequencing the *β‐tubulin* gene, and minimum inhibitory concentrations were determined by broth microdilution. The *cyp51A* gene was sequenced to detect mutations, and CRISPR‐Cas9 genome editing was employed to investigate their influence on susceptibility patterns. Microsatellite genotyping was performed to assess genetic variability, followed by whole genome sequencing and single nucleotide polymorphism analysis.

**Results:**

The environmental strains presented the same *cyp51A* genotype characterised by the M172V substitution and silent mutations. Microsatellite genotyping and whole genome sequencing confirmed that the strains were clonal. Functional validation demonstrated that the M172V and silent mutations partially contribute to reduced susceptibility to tebuconazole but are not the main mechanism of resistance involved. Analysis of polymorphisms in genes other than *cyp51A* revealed no resistance‐conferring mutations.

**Conclusions:**

The findings described herein suggest the possibility of local clonal dissemination of environmental strains under selective pressure from agricultural azoles in a major agribusiness region of the Midwest of Brazil. This study highlights the silent spread of potentially resistant genotypes in urban areas and reinforces the need for environmental surveillance and expanded genomic monitoring in South America.

## 1 Introduction

Aspergillosis is an infection initiated by the inhalation of spores of *Aspergillus* spp., with *A. fumigatus* being the main pathogen, currently listed as a critical priority fungus by the World Health Organization (WHO) [1]. Exposure to spores occurs daily, but disease development depends on the host's predisposing factors, with mortality rates exceeding 50%, especially in immunocompromised, transplant, or cancer patients [2].

The growing interest in *A. fumigatus* is primarily due to its resistance to triazole antifungals, the primary therapeutic class used to treat aspergillosis. Prolonged use of these drugs can select for resistant strains [3]. Additionally, triazole fungicides, also known as Demethylation inhibitors (DMI), are widely used in agriculture, potentially favouring mutations or the selection of better adapted strains. This environmental pressure can lead to cross‐resistance between agricultural and clinical triazoles, as demonstrated by resistant strains found in patients with no prior exposure to antifungal treatment [4]. This theory has been strengthened by genomic studies showing a link between environmental and clinical strains [5].

Brazil is a major agricultural producer, benefiting from diverse species and geographical conditions that favour the occurrence of pathogens, particularly in Mato Grosso do Sul (MS), one of the country's key agricultural regions. According to the Brazilian Institute of Environment and Renewable Natural Resources (IBAMA) in 2023, tebuconazole was the most sold triazole fungicide in MS, with a 100% increase in sales over the past 5 years [6].

Mutations in the *cyp51A* gene, which encodes the triazole target enzyme 14‐α‐demethylase, are the main mechanisms of resistance. These include single nucleotide polymorphisms (SNPs) with or without tandem repeat (TR) insertions in the promoter region [3]. Furthermore, there are combinations of *cyp51A* mutations, *cyp51A*‐5SNPs (F46Y/M172V/N248T/D255E/E427K) and *cyp51A*‐3SNPs (F46Y/M172V/E427K), that are frequently accompanied by silent mutations such as G89G, L358L, C454C, and G497G [7]. Notably, well‐known point mutations such as G54, M220, and G448 are frequently found as single mutations. In contrast, the SNPs that comprise the 3SNPs and 5SNPs profiles are not always seen alone, typically occurring as combined sets [7, 8]. The *cyp51A‐*3SNPs and *cyp51A*‐5SNPs are clustered in a distant clade from the wild‐type (WT) clade, although details about their evolution are unknown [9]. Strains with these combinations tend to have higher minimum inhibitory concentrations (MICs) for triazoles; however, some of these mutations are also present in susceptible strains, raising questions about their role in resistance [7, 8, 10, 11].

In this study, we describe five *Aspergillus fumigatus* strains with reduced susceptibility to tebuconazole, carrying the M172V mutation, which is individually considered a key component of the 3SNPs and 5SNPs, along with four silent mutations (L358L, C454C, G497G, and G89G). We assessed the relationship between these strains through microsatellite analysis and whole genome sequencing. Furthermore, through gene editing, we determined whether these mutations were responsible for the reduced susceptibility to tebuconazole.

## 2 Materials and Methods

### 2.1 Strains

This study reports the results of investigating a possible link between two azole‐resistant clinical *Aspergillus* spp. strains with strains from the environment, resulting in the identification of five azole‐resistant environmental strains.

The clinical isolates from two patients with suspected aspergillosis were received as part of routine microbiological procedures and for deposition in the Culture Collection of Mato Grosso do Sul (CMMS). These isolates were obtained during a surveillance study conducted in 2022 in the state of Mato Grosso do Sul, Brazil, and represented all azole‐resistant clinical isolates received by the collection that year. The isolates studied were deposited under the identifiers CMMS 154 and CMMS 51.

Following the retrospective analysis of patient data, it has been observed that both resided in the same city and lived approximately 3 km apart. To monitor and identify potentially related resistant environmental isolates, air sampling was conducted in the vicinity of the patients' residences in the first half of 2024.

This resulted in the isolation of five *A. fumigatus* strains, all of which showed reduced susceptibility to tebuconazole. The strains were designated CMMS 340, CMMS 341, CMMS 342, CMMS 343, and CMMS 344.

The air sampling was performed in two rounds 15 days apart by exposing disposable Petri dishes containing Dichloran Rose Bengal Chloramphenicol Agar (DRBC) and Sabouraud Dextrose Agar (SDA) with chloramphenicol to the air for approximately 2 h near the two patients' home addresses. Following fungal isolation, the environmental isolates (CMMS 340–344) and the clinical isolate CMMS 154 were identified as *A. fumigatus* by conventional morphological methods and sequencing of the *β‐tubulin* gene [12]. Species‐level identification of isolate CMMS 51 could not be confirmed; therefore, it was classified at the section level (*Fumigati*) and was excluded from molecular analyses.

Metadata of the environmental and clinical strains are detailed in Tables S1 and S2, respectively.

### 2.2 Antifungal Susceptibility Test

Initially, broth microdilution tests were performed with clinical and environmental strains according to the protocol of the European Committee on Antimicrobial Susceptibility Testing (EUCAST) E.DEF 9.4, using the following antifungals: voriconazole, itraconazole, posaconazole, isavuconazole, and tebuconazole (Sigma‐Aldrich, St. Louis, MO, USA) [13]. Susceptibility classification was performed according to EUCAST clinical breakpoints [14]. Subsequently, assays with the strains used for *cyp51A* gene transformation were conducted with tebuconazole in another laboratory, following the Clinical and Laboratory Standards Institute (CLSI) M38‐Ed3 broth microdilution method protocols [15]. All tests were performed in duplicate. Additionally, tests with the transformed strains were carried out using pre‐prepared dry plates (Eiken Chemical Co., Tokyo, Japan) with an inoculum of 2.5 × 10^4^ CFU/mL, in accordance with the manufacturer's recommendations [9].

Due to the absence of established breakpoints or epidemiological cut‐off values (ECVs) for tebuconazole, we categorised a strain as resistant when its MIC was > 4 μg/mL, and the inhibition concentrations (IC100) were evaluated, as previously proposed [16, 17].

### 2.3 Disk Diffusion Test

Disk diffusion test for tebuconazole was conducted according to CLSI M51‐A Disk diffusion and Qiao et al. (2007) slightly modified [18, 19]. To this end, 20 mL of Mueller‐Hinton agar medium was poured into a plate and mixed with 200 μL of 0.05% Tween 20 containing 2.0 × 10^5^ spores. After the medium solidified, a 6 mm disk containing 10 μg of tebuconazole was placed on the surface. The plates were incubated at 35°C for 40 or 48 h, and the diameters of the inhibition zones were measured. All measurements (mm) for each transformation were performed in duplicate.

### 2.4 Sequencing of *cyp51A* Regions

Genomic DNA was extracted from overnight‐cultured mycelia using the phenol‐chloroform method [20].

Sequencing of the *cyp51A* coding sequence, as well as its upstream and downstream flanking regions, was carried out using specifically designed primers (Table S3), following protocols previously described [9]. Nucleotide sequences of the DNA fragments were determined using BigDye Terminator v1.1/3.1 (Applied Biosystems) and an ABI automated sequencer (PerkinElmer). Sequence alignments were then performed against the reference sequence (A1163, FungiDB) using the Benchling platform (https://www.benchling.com/).

The sequence data of the *cyp51A* mutations generated in this study are available in GenBank under accession numbers PX655623, PX655624, PX655625, PX655626, and PX655627.

### 2.5 Microsatellite (Short Tandem Repeats (STRs)) Analysis

The microsatellite (STR) analysis was performed using nine markers using the primers described in the Table S3. The repeat numbers were determined at the specific loci: 2A, 2B, 2C, 3A, 3B, 3C, 4A, 4B, and 4C based on the sequencing data [21]. The tandem repeats were counted and analysed using SnapGene Viewer software. A dendrogram was constructed employing the UPGMA algorithm with a minimum spanning tree approach in the BioNumerics software version 7.6 (Applied Math Inc., Austin, TX, USA). The references for the other strains used to construct the dendrogram are listed in Table S4.

### 2.6 Whole Genome Sequencing and Analysis

Genomic DNA was extracted from overnight‐cultured mycelia using the Quick‐DNA Fungal/Bacterial Miniprep Kit (Zymo Research, USA). Whole genome sequencing was performed for the five environmental strains. Sequencing libraries were prepared using the *DNA Library Prep Kit with Fragmentation* (WatchMaker Genomics, Beverly, MA, USA) following the manufacturer's protocol. Paired‐end sequencing (2 × 150 bp) was performed on an Illumina NovaSeq X platform at the Translational Genomics Research Institute (TGen; Phoenix, AZ, USA), targeting a minimum of 100× genome coverage per strain.

Initially, the raw whole genome sequencing data were trimmed for Illumina adapters using the fastp v0.23.4 software [22]. Subsequently, the five genomes were assembled *de novo* using SPAdes v3.15.5 [23]. Average Nucleotide Identity (ANI) analysis was performed using the pyani v0.3.0 tool, mANI method, comparing all strains to each other, including the Af293 genome as a reference for *A. fumigatus* .

Mating type identification was performed by mapping the assembled contigs to the reference sequences of the genes *MAT1‐1‐1* (AY898661), *MAT1‐2‐1* (Afu3g06170), and *MAT1‐2‐4* (Afu3g06160) using minimap2 v.2.30 [24]. After mapping, coverage was calculated with SAMtools v1.22. Genes with coverage greater than 99% were used to confirm the presence of the mating‐type (*MAT*) genes. The presence of the *MAT1‐1‐1* gene was used to confer MAT1‐1, while the presence of the *MAT1‐2‐1* and *MAT1‐2‐4* genes to confer MAT1‐2 as mating type [25].

The sequencing reads and assemblies have been deposited at DDBJ/ENA/GenBank under the PRJNA1354845 project number.

### 2.7 Single Nucleotide Polymorphism Analysis

The genome of *A. fumigatus* A1163 strain (GCA_000150145.1) was used as reference. The reference sequence was prepared for downstream analyses by indexing with BWA index, SAMtools faidx (v1.22) [26] and GATK CreateSequenceDictionary (v4.6.2.0) [27]. Trimmed reads were mapped to A1163 using BWA‐MEM (v0.7.19) [28]. The resulting alignments were sorted by coordinate with SAMtools sort, and read groups were added using Picard AddOrReplaceReadGroups (v3.4.0) to ensure correct sample labelling. PCR duplicates were marked using Picard MarkDuplicates. The alignment files were indexed using SAMtools index.

Variant calling was performed to detect SNPs and indels. Variants were output in Variant Call Format (VCF), including the Genomic VCF (GVCF) intermediate. GATK HaplotypeCaller was used in ERC GVCF mode to generate per‐sample GVCFs. Allele balance for homozygous calls (ABHom) was calculated with AddAlleleBalance from VCFProcessor (v1.2.6). Individual GVCF files were indexed, combined into a single cohort GVCF using GATK CombineGVCFs, and jointly genotyped with GATK GenotypeGVCFs.

To ensure high‐quality variants, low‐confidence calls were filtered using GATK VariantFiltration and SelectVariants, based on the following metrics from Rhodes et al. [5], depth of coverage (DP) < 10, Mapping Quality (MQ) < 40.0, Quality by Depth (QD) < 2.0, Fisher Strand (FS) > 60.0, ABHom < 0.9, Genotype Quality (GQ) < 50.0.

Functional annotation of high‐quality SNPs and indels was performed using SnpEff (v5.3.0a‐1) [29]. Annotated variants within *cyp51A* (AFUB_063960), *cyp51B* (AFUB_089270), *hapB* (AFUB_030360), *hapC* (AFUB_004250), *hapE* (AFUB_092980), *hapX* (AFUB_0524200), *hmg1* (AFUB_020770), *cox7c* (AFUB_064240), *cox10* (AFUB_065450), *NctA* (AFUB_029870) and *NctB* (AFUB_045980) were extracted for further analysis.

### 2.8 Single‐Guide RNA Synthesis and Repair Template Construction

The guide RNA (sgRNA) was constructed according to the Umeyama protocol [30]. For the transformation experiment to verify which SNP is involved in tebuconazole resistance, three repair templates were constructed using the Umeyama method, based on sewing and overlap extension PCR [30]. The primers used to construct the repair template are shown in Table S3; they include the upstream and downstream regions of *cyp51A*, as well as silent mutations, which were inserted into the fragment to confer resistance to Cas9 nuclease. Additionally, a hygromycin B resistance marker cassette (*hph*) obtained from the pHph plasmid was also included in the plasmid construction.

The first template was constructed by cloning the *cyp51A* region amplicons from strain (CMMS 340) and fusing them with *hph* into the pBluescript II SK(+) vector, thereby introducing the mutations M172V, G89G, L358L, C454C, and G497G (*cyp51A* ^340^). The overlap and sewing PCR used to fuse the fragments was performed using the Q5 Hot Start High‐Fidelity 2× Master Mix (New England Biolabs). Then, the fragments were amplified.

The second and third repair templates were constructed from the AfS35 strain to generate a wild‐type mutant (*cyp51A* ^WT^), one containing only the M172V mutation (AfS35^M172V^) without silent mutations. They were amplified through inverse PCR with the plasmid carrying *cyp51A* ^WT^, which was previously constructed according to Majima (2021) [9].

After the amplicons were fused, the fragments were purified using the FavorPrep kit and then cloned into the pBluescript II SK(+) vector, which had been digested with *HindIII* and *KpnI*, using the In‐Fusion HD Cloning Kit (TAKARA Bio, Shiga, Japan), in *Escherichia coli* (Nippon Gene Ecos DH5α).

### 2.9 *Aspergillus fumigatus* Transformation

Transformation was performed by homologous recombination using the protoplast‐polyethylene glycol method according to Umeyama [30]. Prior to the transformation, spores from strains CMMS 340 and AfS35 were incubated in YG medium for 6 h at 37°C for protoplast preparation. They were then incubated in 0.2 g/mL Vino‐Taste Pro (Novozymes, Denmark) for 90 min at 30°C.

A mixture of 10 μg of HiFi Cas9 Nuclease V3 (Integrated DNA Technologies, Coralville, IA) and 10 pmol of each sgRNA (sgRNA1 and sgRNA2) was incubated at room temperature for 25 min and combined with the repair templates before being added to the protoplasts. After transformation, the protoplasts were plated onto Czapek Dox agar supplemented with 1 M sucrose. Following 20 h of incubation at 37°C, a top layer of agar containing 400 μg/mL of hygromycin was added to select for transformants. The resulting colonies were then collected and subcultured.

## 3 Results

### 3.1 Clinical Strains Data and Air Sampling

The CMMS 154 strain was isolated from a sputum sample and, based on diagnosis, corresponded to a probable case of chronic pulmonary aspergillosis, showing resistance to voriconazole (4 mg/L), itraconazole (> 16 mg/L), and isavuconazole (2 mg/L).

The CMMS 51 isolate, categorised as *Aspergillus* spp. from section *Fumigati*, was recovered from a tracheal aspirate and was considered a case of colonisation. It showed resistance to voriconazole (4 mg/L) and isavuconazole (8 mg/L). As the strain could not be confirmed as *A. fumigatus* , it was excluded from further analyses.

In attempt to link these clinical strains with the population present in the patients' environment two rounds of air sampling were conducted in the vicinity of the patients' homes, resulting in the isolation and identification of five *A. fumigatus* strains resistant to tebuconazole.

### 3.2 Microsatellite (STR) Analysis

**FIGURE 1:**
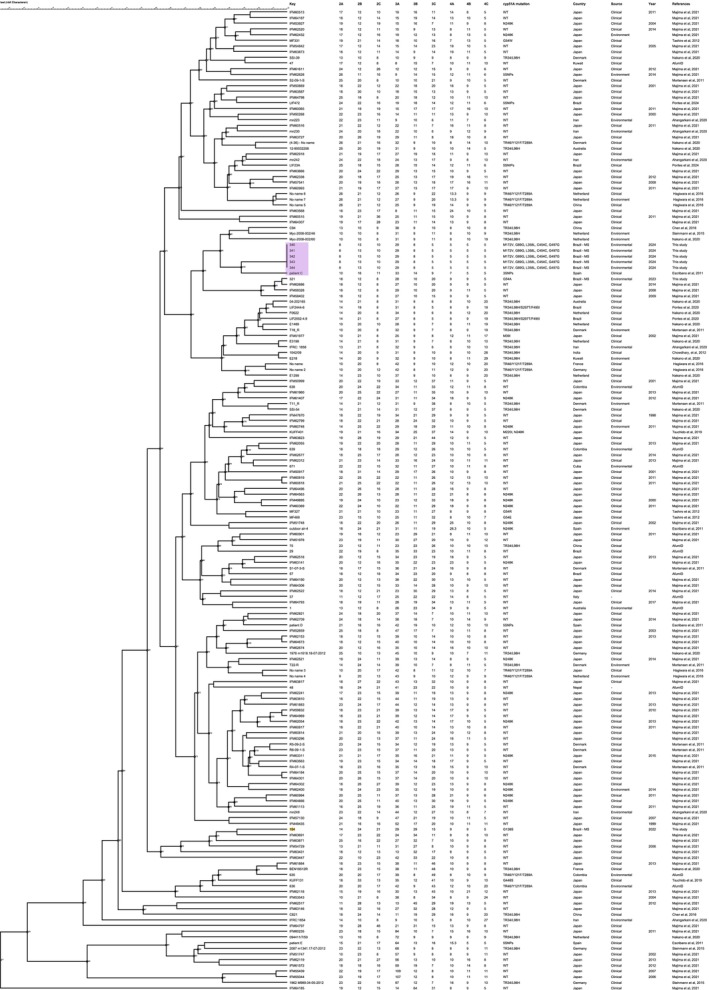
Dendrogram constructed using the Unweighted Pair Group Method With Arithmetic Mean (UPGMA) and Minimum Spanning Tree (MST) based on Short Tandem Repeat (STR) profiles.

STR analysis showed that the five environmental strains clustered in the same clade as a strain from Spain (referred to as Patient C) carrying three SNPs in *cyp51A*. The CMMS 154 strain clustered separately from the others (Figure 1). Based on this result,no association was observed between clinical and environmental strains.

### 3.3 Azole Susceptibility Testing and *cyp51A* Genotype

All five airborne strains collected from the vicinity of the patients' residence exhibited resistance to tebuconazole (8 mg/L) and carried five polymorphisms, including one missense mutation (M172V) and four silent mutations (L358L, C454C, G497G, and G89G), Table S5. In contrast, the clinical strain CMMS 154 carried a G138S mutation as presented in Table 1.

**TABLE 1:** Minimum inhibitory concentration values and *cyp51A* genotype of *Aspergillus fumigatus* strains.

STRAIN ID^a^	Clinical triazoles MIC				DMI	*cyp51A* mutations
	VOR	ISA	ITC	POS	TEB	
CMMS 154	**4**	**2**	**> 16**	0.03	4	G138S
CMMS 340	1	1	1	0.25	**8**	M172V, L358L, C454C, G497G, G89G
CMMS 341	0.5	0.25	1	0.25	**8**	M172V, L358L, C454C, G497G, G89G
CMMS 342	1	1	0.5	0.25	**8**	M172V, L358L, C454C, G497G, G89G
CMMS 343	0.5	0.25	1	0.25	**8**	M172V, L358L, C454C, G497G, G89G
CMMS 344	0.5	0.25	0.5	0.25	**8**	M172V, L358L, C454C, G497G, G89G

*Note:* MIC was performed following the European Committee on Antimicrobial Susceptibility Testing–EUCAST protocol.

^a^ Strain CMMS 154 refers to clinical isolate; Strains CMMS 340 to CMMS 344 are airborne strains collected around the patients' houses; CMMS = culture collection of Mato Grosso do Sul; ISA = isavuconazole; ITC = itraconazole; POS = posaconazole; TEB = tebuconazole; VOR = voriconazole.

### 3.4 Whole Genome Sequencing

The genomes of CMMS 340–344 strains showed high similarity with at least 99.99% ANI (Table S6) with the ANI analysis covering 99.88% of the individual genomes (Table S7). Furthermore, the investigation of the mating types established that all five environmental strains belong to the MAT1‐2 mating type.

### 3.5 Polymorphisms in Non‐ *cyp51A* Genes

Polymorphisms in *cyp51B*, *hapB*, *hapC*, *hapE*, *hapX*, *hmg1*, *cox7c*, *cox10*, *NctA*, and *NctB* were examined to assess variation in additional azole‐associated genes. An overview of the identified polymorphisms is presented in Table 2. No previously described resistance‐associated polymorphisms were identified in these genes.

**TABLE 2:** Polymorphisms identified in non‐*cyp51A* genes associated with azole resistance.

Gene	CMMS 340—CMMS 344	CMMS 154
*cyp51B*	WT	WT
*hapB*	WT	H275N
*hapC*	WT	WT
*hapE*	WT	WT
*hapX*	I96V/E194D/T461N	WT
*hmg1*	H564Y	WT
*cox7c*	L130W/F136S	WT
*cox10*	WT	WT
*NctA*	V72I	WT
*NctB*	WT	WT

Abbreviation: WT, wild‐type.

### 3.6 Tebuconazole Testing of the Transformant of *A. fumigatus* Strain CMMS 340

The AfS35‐derived transformant (AfS35^340^) showed a one‐dilution increase in MIC, while no MIC change was observed for the AfS35^M172V^ genotype. The susceptibility test results for the strains before and after transformation are shown in Table 3.

**TABLE 3:** Minimal inhibitory concentration values from the transformant strains of *Aspergillus fumigatus* and their respective genotypes performed through Clinical & Laboratory Standards Institute–CLSI protocol.

Strains	Genotype	TEB	VOR	ITC
		IC100		
Background CMMS 340	*cyp51A* ^340^	8	2	1
*cyp51A* ^340^	Δ*cyp51A* ^340^:: *cyp51A* ^340^:: *hph*	8	2	1
*cyp51A* ^WT^	Δ*cyp51A* ^340^:: *cyp51A* ^WT^:: *hph*	8	2	1
Background AfS35	*cyp51A* ^WT^	2	1	0.5
AfS35^WT^	Δ*cyp51A* ^WT^:: *cyp51A* ^WT^:: *hph*	2	ND	ND
AfS35^340^	Δ*cyp51A* ^WT^:: *cyp51A* ^340^:: *hph*	4	ND	ND
AfS35^M172V^	Δ*cyp51A* ^WT^:: *cyp51A* ^M172V^:: *hph*	2	ND	ND

Abbreviations: ITC, itraconazole; ND, not determined; TEB, tebuconazole; VOR, voriconazole.

In comparison, the CMMS 340 background conferred resistance to tebuconazole (MIC > 4) and voriconazole (MIC ≥ 2) irrespective to the *cyp51A* genotype tested.

The disk diffusion test results confirmed the findings from the susceptibility tests, showing a slight decrease in susceptibility to tebuconazole in the transformant strains compared to the parental strain AfS35. Two strains recovered from the transformed strain AfS35 *cyp51A* ^340^ (AfS35^340‐1^, AfS35^340‐5^) and AfS35 (AfS35^M172V‐4^, AfS35^M172V‐5^) were tested. The halo diameter results are described in Table 4.

**TABLE 4:** Disk diffusion zone diameters (expressed in mm) for parental and transformant *Aspergillus fumigatus* strains under tebuconazole exposure after 24, 40 and 48 h.

Genotype	24 h	Average	40 h	Average	48 h	Average
AfS35^WT^	34	33.3	35	35	34	34.3
	32		35		33	
	34		35		36	
AfS35^340‐1^	29	30.5	30	31.5	30	31
	30		30		30	
AfS35^340‐5^	32		33		32	
	31		33		32	
AfS35^M172V‐4^	29	31	33	32.5	32	31.2
	31		32		31	
AfS35^M172V‐5^	32		32		31	
	32		33		31	

## 4 Discussion

This study describes the detection of five airborne *A. fumigatus* strains resistant to tebuconazole in the vicinity of two patients suspected of having aspergillosis. The strains were isolated via an air sampling study in an attempt to link the clinical strains CMMS 51 and CMMS 154 to environmental *A. fumigatus* strains circulating in the air. Based on the obtained sequence data, the two clinical strains could not be linked to the five environmental strains. Here we present an in‐depth investigation of *cyp51A* sequence variation underlying the resistance to tebuconazole of these environmental strains.

Tebuconazole is a triazole fungicide from the group of Demethylation Inhibitors (DMI), with a short side chain, structurally similar to the voriconazole molecule [31]. Although the In Vitro selection of resistant strains has been documented in previous studies [32, 33], the mechanisms driving resistance in environmental settings are likely to be more complex. Worsening the situation, pesticide use in developing countries often suffers from poor regulation, limited oversight, and reliance on outdated compounds [34]. Fungicides have a long half‐life, and during spraying, more than 50% of the initial dose may be lost [35]. As a result, these molecules can travel through the air and reach areas beyond the application site. Likewise, conidia may disperse over long distances depending on weather conditions [36]. The contamination of water with pesticides, including tebuconazole, in the central‐west region of Brazil, the same region where the state of MS is located, has already been reported, indicating excessive usage [37, 38].

The five strains came from the outskirts of an urban area. There were no nearby flowerbeds or known sources of fungicide exposure, leaving the origin of these strains unclear. Previous studies have reported clinical strains acquiring resistance in the environment before infecting azole‐naive patients [4, 5]. The strains in the current study appear to follow a similar pattern, likely developing environmental resistance to tebuconazole, which subsequently reduced their susceptibility to voriconazole. A study by Sewell et al. [39] found more resistant environmental strains of *A. fumigatus* in urban areas (13.8%) than in rural areas (1.1%). It is hypothesised that the high genetic diversity in urban centres may increase the evolutionary potential, since resistance alleles that have been introduced into the city have the opportunity to integrate into new genetic backgrounds through recombination. Another reason may be the presence and distribution of horticultural crops in cities, such as flowers, ornamental plants, and vegetables, which may require the use of fungicides [39]. The strains reported in this study were collected on different days in a distance of 3 km, and since aspergillosis is typically acquired through inhalation of airborne spores, the presence and circulation of such resistant strains in urban areas raises significant concern.

Sequencing of the *cyp51A* gene in the five strains resistant to tebuconazole revealed an M172V polymorphism, accompanied by four additional silent mutations (L358L, C454C, G497G, and G89G). The M172V mutation is one of those that compose the *cyp51A*‐3SNPs (F46Y/M172V/E427K) and 5SNPs (F46Y/M172V/N248T/D255E/E427K) haplotypes [8]. The occurrence of one of these mutations alone, without the full combination of 3SNPs to 5SNPs, has been reported in the literature and is less frequent, as in the case of a strain from Nigeria, isolated from a flowerbed, which also carried only the M172V mutation and showed increased MICs for both tebuconazole and itraconazole [16]. In addition, a previous study in Brazil described seven clinical strains with only the M172V substitution, six of which had reduced susceptibility for itraconazole, voriconazole and posaconazole, alternatively, and one susceptible, but data for tebuconazole for those strains were not available [40].

The CMMS 154 isolate carried a G138S mutation in *cyp51A*, which has been associated with resistance to triazoles [41, 42]. This mutation has also been previously described not only in clinical isolates but also in soil isolates associated with resistance to the DMI fungicide propiconazole [42].

The functional role of the identified mutations was validated through precise genomic editing using the CRISPR‐Cas9 system. In the susceptible AfS35 strain, the insertion of *cyp51A* ^340^ allele resulted in a one‐dilution increase in the tebuconazole MIC, whereas the restoration of the wild‐type allele *cyp51A* ^WT^ in the CMMS 340 strain didn't increase its susceptibility. These findings indicate that although these mutations possess an inherent capacity to reduce susceptibility, their impact is strictly modulated by the genomic context. This suggests that in a strain like CMMS 340, the resistance phenotype is robust and multifactorial.

Among the genes analysed, most were wild‐type. Furthermore, the identified mutations have not been associated with the resistance phenotype, although these genes have been previously linked to triazole resistance. Three polymorphisms were found in the *hapX* gene, but they were located outside the binding motif. Normally, *HapX* functions as a repressor of *cyp51A* gene transcription. Mutations in the *HapX* DNA‐binding motif prevent its proper binding to DNA, resulting in loss of repression and increased *cyp51A* expression, which can lead to azole resistance [43, 44].

*Hmg1* encodes a 3‐hydroxy‐3‐methylglutaryl‐CoA (HMG‐CoA) reductase that catalyses the conversion of HMG‐CoA to mevalonic acid and acts as the rate‐limiting enzyme in ergosterol biosynthesis [45, 46]. Mutations in *hmg1*, particularly within the Sterol‐Sensing Domain (SSD), have been associated with azole resistance, either alone or in combination with *cyp51A* mutations [45, 46]. However, the H564Y substitution is located outside the SSD and has also been reported in a susceptible strain [47].

Chen et al. [48] described a W56* mutation in the *cox7c* gene and validated its function through targeted gene deletion. This gene encodes subunit VIIc of cytochrome c oxidase, an enzyme involved in the mitochondrial respiratory chain in eukaryotes. Loss of its mitochondrial function reduced the production of reactive oxygen species (ROS), among other effects, conferring multidrug resistance to the fungus. However, other mutations, such as L130W and F136S, have not yet been associated with resistance.

*NctA* encodes a subunit of the Negative Cofactor 2 (NCT) complex, which acts as a global transcriptional co‐regulator in the fungus [49]. Functional studies have shown that loss of *NctA* alters the expression of multiple genes and increases ergosterol levels, resulting in high levels of resistance [49]. The mutation (V72I) observed in this study is unlikely to result in a loss of function of this gene.

These findings suggest that the polymorphisms identified in non‐*cyp51A* genes are unlikely to contribute to the tebuconazole resistance phenotype observed. One possible alternative mechanism is the overexpression of efflux pumps, which plays an important role in triazole resistance in fungi, including *A. fumigatus* [50, 51]. Therefore, approaches that evaluate the upregulation of these genes at the transcriptomic level, as previously described for genes such as *atrF, abcB, mdr3*, and *mdr1*, are essential [48]. The influence of the mutations that make up the 3SNPs and 5SNPs has shown conflicting evidence, as they are present in both susceptible and resistant *A. fumigatus* [8]. In our strains, we observed that M172V, accompanied by four additional silent mutations, partially contributes to reducing susceptibility to tebuconazole but does not fully explain the observed resistance. In the future, further studies with these strains may elucidate the mechanisms that operate in conjunction to contribute to resistance to triazoles.

Furthermore, none of the herein studied strains carried the TR mutations previously associated with tebuconazole resistance [4, 33]. Toyotome et al. [52], through in vitro selection, observed the emergence of mutations in various genes but were unable to induce the classic alterations in *cyp51A* or TR insertions in the promoter, as previously described [31]. Non‐*cyp51A* genes were involved in resistance to tebuconazole, which is most likely the case for the herein studied strains.

The microsatellite profiles of the strains showed that the five strains, beyond sharing the same phenotype along with being resistant to tebuconazole, are from the same genetic group. Although they were collected at different sites and on different days, they were all from the same neighbourhood. The sample size is too small to draw broad conclusions; however, it is possible that resistant strains are being dispersed into the region from external sources. The identification of these resistant isolates circulating in urban environments raises considerable concern and highlights the importance of continuous surveillance in these areas. Whole genome sequencing analyses corroborate the microsatellite findings, as the ANI analysis revealed high genomic identity among the environmental strains (CMMS 340—CMMS 344). In addition, all five strains belong to the MAT1‐2 mating‐type. The short tandem repeat profile and the *cyp51A* mutations found confirm that there is no epidemiological link between the clinical strain CMMS 154 and the environmental strains.

*Aspergillus fumigatus* carrying triazole resistance polymorphisms has been shown in previous studies to exhibit a clear population structure, are non‐randomly distributed, have low genetic diversity, are closely related, and exhibit rapid selection for beneficial mutations that promote global expansion and clonal reproduction [5, 53]. Additionally, some *cyp51A* mutations reported in the literature do not appear to affect fitness or morphotype, potentially facilitating their persistence in *A. fumigatus* populations without a significant selective cost [54].

The dendrogram constructed from microsatellite markers showed that the five environmental strains in this study clustered together with a strain called ‘Patient C’ from a Spanish study by Escribano et al. [8]. Notably, the Patient C strain is clinical and possesses the 3 SNPs (F46Y/M172V/E427K) and the silent mutations (L358L, C454C, G497G, G89G), suggesting a common evolutionary origin.

Future studies, including a larger collection of isolates, coupled with whole genome and expression analyses, will be essential to clarify additional resistance mechanisms involved in these strains and to better understand their phylogenetic relationships. This study provides evidence of the environmental circulation of *A. fumigatus* strains resistant to tebuconazole in an urban setting in Brazil. These strains raise concern about the clonal dissemination of resistance through environmental routes.

## 5 Conclusion

This study brings evidence of environmental circulation of *A. fumigatus* strains resistant to tebuconazole in an urban setting in Brazil. These strains raise concern about the clonal dissemination of resistance via environmental routes. The findings reinforce the need for ongoing environmental monitoring and underscore the importance of considering non‐clinical reservoirs in antifungal resistance research. Further genomic studies are needed to trace the origin and dissemination routes of these resistant isolates.

## Author Contributions

**Akira Watanabe:** conceptualisation, investigation, methodology, validation, data curation, writing – review and editing, project administration, resources. **Teppei Arai:** conceptualisation, writing – review and editing, data curation, methodology, formal analysis. **Wellington Santos Fava:** investigation, methodology. **Marcia de Souza Carvalho Melhem:** funding acquisition, conceptualisation, methodology, writing – review and editing, project administration, data curation. **Hidetaka Majima:** conceptualisation, investigation, writing – review and editing, data curation, methodology, formal analysis, visualisation, validation, supervision. **Dality Keffelen de Barros Rodrigues:** conceptualisation, investigation, writing – original draft, methodology, validation, visualisation, formal analysis, data curation. **Manuel Leeuwerik:** formal analysis, writing – review and editing, data curation. **Wieland Meyer:** data curation, writing – review and editing, methodology, resources. **Balázs Brankovics:** formal analysis, writing – review and editing, data curation. **James Venturini:** writing – review and editing, resources.

## Funding

This research was supported by National Council for Scientific and Technological Development (CNPq) (Grants 443813/2023‐0 and 201422/2024‐7); Coordination for the Improvement of Higher Education Personnel (CAPES) (Grants 88887.658397/2021‐00 and 88887.819536/2023‐00).

## Consent

This study was approved by the Human Ethics Committee (approval number CAAE: 59722522.0.0000.0021).

## Conflicts of Interest

The authors declare no conflicts of interest.

## Supporting information


**Table S1:** Metadata of air sampled *Aspergillus fumigatus* isolates.

**Table S2:** Metadata of clinical samples.

**Table S3:** List of primers used in this study.

**Table S4:** Short tandem repeat (STR) profiles and corresponding references.

**Table S5:** Nucleotide changes and polymorphisms in *cyp51A* of air sampled *Aspergillus fumigatus* isolates.

**Table S6:** Average Nucleotide Identity percentage among *Aspergillus fumigatus* air isolates.

**Table S7:** Genome alignment coverage among *Aspergillus fumigatus* air isolates.

## Data Availability

The data that support the findings of this study are openly available in the National Center for Biotechnology Information (NCBI), under the following accession numbers: BioProject PRJNA1354845, BioSample, SAMN52959256–SAMN52959260, Sequence Read Achive (SRA) SRR36017302‐SRR36017306, Whole genome assemblies JBSKJA000000000–JBSKJE000000000 and GenBank PX655623‐PX655627.
